# Stem Cell Antigen-1 (Sca-1) Regulates Mammary Tumor Development and Cell Migration

**DOI:** 10.1371/journal.pone.0027841

**Published:** 2011-11-29

**Authors:** Torey D. Batts, Heather L. Machado, Yiqun Zhang, Chad J. Creighton, Yi Li, Jeffrey M. Rosen

**Affiliations:** 1 Interdepartmental Program in Cell & Molecular Biology, Baylor College of Medicine, Houston, Texas, United States of America; 2 Department of Molecular and Cellular Biology, Baylor College of Medicine, Houston, Texas, United States of America; 3 Dan L. Duncan Cancer Center at Baylor College of Medicine, Houston, Texas, United States of America; 4 Lester & Sue Smith Breast Center, Baylor College of Medicine, Houston, Texas, United States of America; The University of Texas M.D. Anderson Cancer Center, United States of America

## Abstract

**Background:**

Stem cell antigen-1 (Sca-1 or Ly6A) is a glycosyl phostidylinositol (GPI)-anchored cell surface protein associated with both stem and progenitor activity, as well as tumor initiating-potential. However, at present the functional role for Sca-1 is poorly defined.

**Methodology/Principal Findings:**

To investigate the role of Sca-1 in mammary tumorigenesis, we used a mammary cell line derived from a MMTV-Wnt1 mouse mammary tumor that expresses high levels of endogenous Sca-1. Using shRNA knockdown, we demonstrate that Sca-1 expression controls cell proliferation during early tumor progression in mice. Functional limiting dilution transplantations into recipient mice demonstrate that repression of Sca-1 increases the population of tumor propagating cells. In scratch monolayer assays, Sca-1 enhances cell migration. In addition, knockdown of Sca-1 was shown to affect cell adhesion to a number of different extracellular matrix components. Microarray analysis indicates that repression of Sca-1 leads to changes in expression of genes involved in proliferation, cell migration, immune response and cell organization.

**Conclusions/Significance:**

Sca-1 exerts marked effects on cellular activity and tumorgenicity both *in vitro* and *in vivo*. A better understanding of Sca-1 function may provide insight into the broader role of GPI-anchored cell surface proteins in cancer.

## Introduction

Stem cell antigen-1 (Sca-1 or Ly6A) is a member of the Ly6 family of glycosyl phostidylinositol (GPI)-anchored cell surface proteins. Sca-1 has been long associated with murine stem/progenitor cells [1] and is localized to lipid rafts where it regulates signaling complexes [2]. Functional studies using Sca-1-null mice have revealed several phenotypes. Interferon-stimulated hematopoietic stem cells (HSCs) upregulate Sca-1 in a Stat1-dependent manner. Additionally, minor defects in lineage skewing were observed in the hematopoietic system of Sca-1-null mice. Osteoporosis and reduced muscle size were observed in aging Sca-1-null mice. Moreover, Sca-1 is necessary for matrix metalloproteinase (MMP) activity during muscle repair.

Initial studies in the mammary gland showed that Sca-1^+^ cells have increased regenerative capacity compared to Sca-1^−^ cells [3]. Subsequent studies involving purified mammary stem cells with repopulating activity using CD24 in combination with CD29 (β1 integrin) or CD49f (α6 integrin) indicated that these cells express low levels of Sca-1 [4], [5]. Instead, CD24^high^ luminal progenitor cells were shown to differentiate into Sca-1^+^ estrogen receptor (ER) expressing cells and Sca-1^−^/ER^−^ cells [6].

When Sca-1 or other Ly6 family members are upregulated on tumor cells they are commonly associated with an aggressive phenotype [7]. Sca-1^+^ cells are expanded in mammary tumors induced by Wnt/β-catenin pathway [8], [9]. Despite its association with stem/progenitor cells, little is known about the biological function of Sca-1. To address this question in the context of mammary tumor development, we used a cell line derived from primary tumors of MMTV-Wnt1 transgenic mice, which retained high expression of Sca-1 and could be transplanted into the cleared fat pad of syngenic mice. We found that Sca-1 promotes cell migration and decreases cell adhesion *in vitro* and regulates tumorigenicity upon transplantation. Furthermore, Sca-1 regulates gene expression in multiple pathways involved in tumor progression. This study demonstrates that modulating Sca-1 expression has profound effects on cellular function and tumor development.

## Results

### Sca-1 promotes cell migration

**Figure 1:**
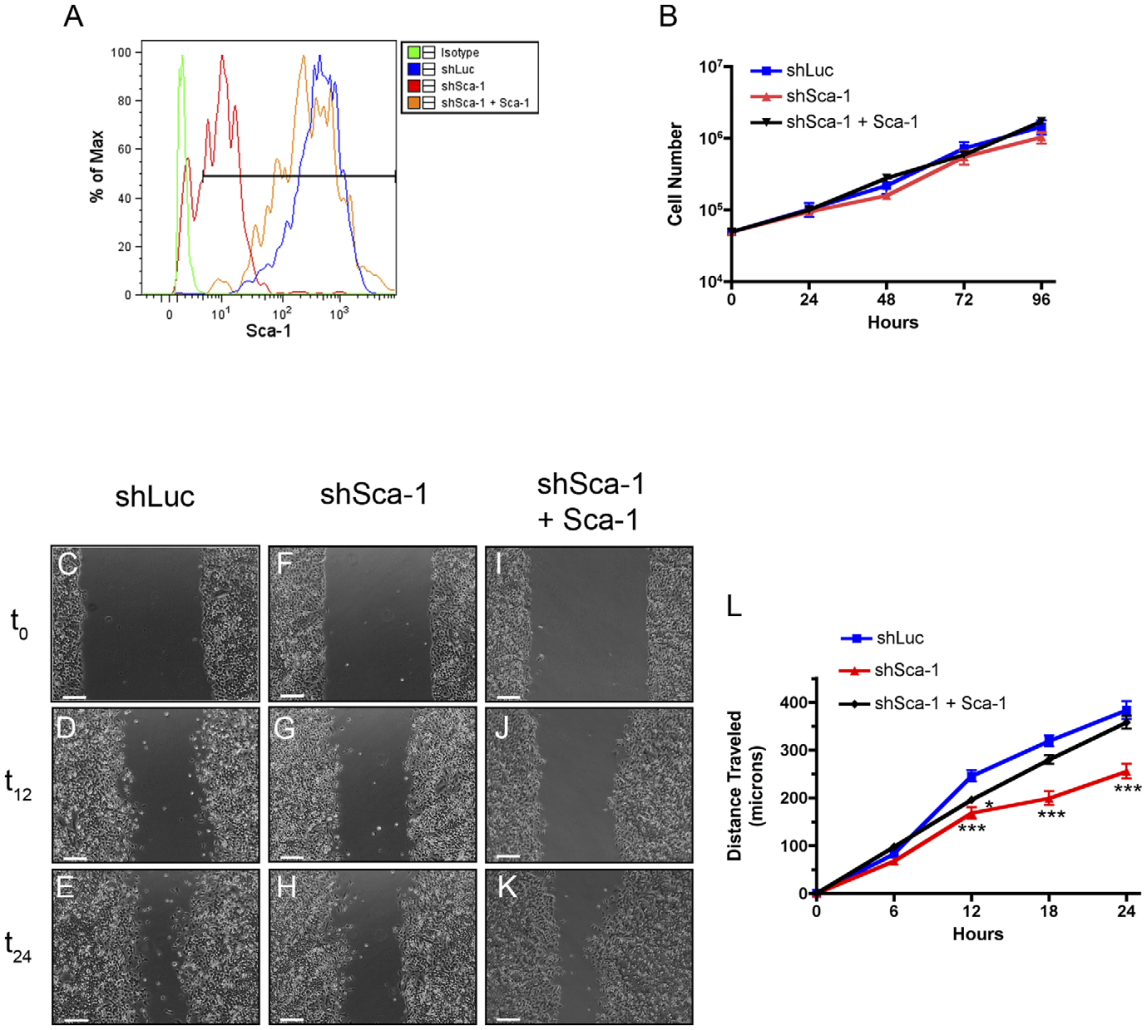
Repression of Sca-1 delays cell migration. Flow cytometry analysis of Sca-1 surface expression, representative histograms (A). Growth curve of shLuc (blue), shSca-1 (red) and shSca-1+Sca-1 (black) cells (B). Images of scratch monolayer migration assay at times 0, 12, and 24 hours (C–K), representative images of 3 experiments performed in triplicate. shLuc (C–E), shSca-1 (F–G), and shSca-1+Sca-1 (I–K), scale bars = 200 µm. Cell migration graph (L). shLuc (blue), shSca-1 (red), shSca-1+Sca-1 (black), * represents statistical significance compared to shLuc control, (* = p<.05, *** = p<.001).

Sca-1 is localized to lipid rafts [2] similar to urokinase plasminogen activator receptor (UPAR), another well-characterized Ly6 family member. UPAR regulates adhesion, migration and angiogenesis in breast cancer [10]. Therefore, we asked whether Sca-1 regulates cell migration using a mammary tumor cell line (Wnt1-YL), derived from primary MMTV-Wnt1 tumors. Previous studies [4], [8] revealed high levels of Sca-1 expression in MMTV-Wnt1 induced hyperplasia and tumors, and we were able to develop several cell lines from these tumors. The Wnt1-YL cells uniformly express high levels of Sca-1 as detected by flow cytometry (Figure 1A). We then knocked down Sca-1 surface expression using shRNA lentiviral technology. A shift in mean fluorescence intensity revealed Sca-1 surface expression was reduced ∼30-fold in the Wnt1-YL-shSca1 (shSca-1) as compared to control cells transduced with an shRNA targeting luciferase (shLuc) (Figure 1A). This reduction in Sca-1 expression did not alter cell growth as assessed by a growth curve over the period of 4 days (Figure 1B). When cell migration was assessed using a wound healing scratch monolayer assay, shSca-1 cells exhibited a significantly slower cell migration at 12–24 hours, (Figure 1C and D). A rescue experiment was next performed by re-introduction of a Sca-1 expression construct containing an altered shRNA-binding site, to rule out off-target effects of the Sca-1 shRNA. Re-expression of Sca-1 reversed the migration phenotype, demonstrating the specificity of the shRNA knockdown (Figure 1C and D). This was also demonstrated independently by microarray analysis in which Ly6a, but not other Ly6 family members, was selectively knocked down by these shRNAs (Table S1). An early lag phase between 0–12 hours was observed in these rescue experiments where there is a significant difference between the shLuc control cells and the shSca-1+Sca-1 rescued cells; however, this delay was overcome by 18 hours. Notably, the cells appeared to migrate collectively as a sheet of cells rather than as single cells.

### Sca-1 regulates cell adhesion

**Figure 2:**
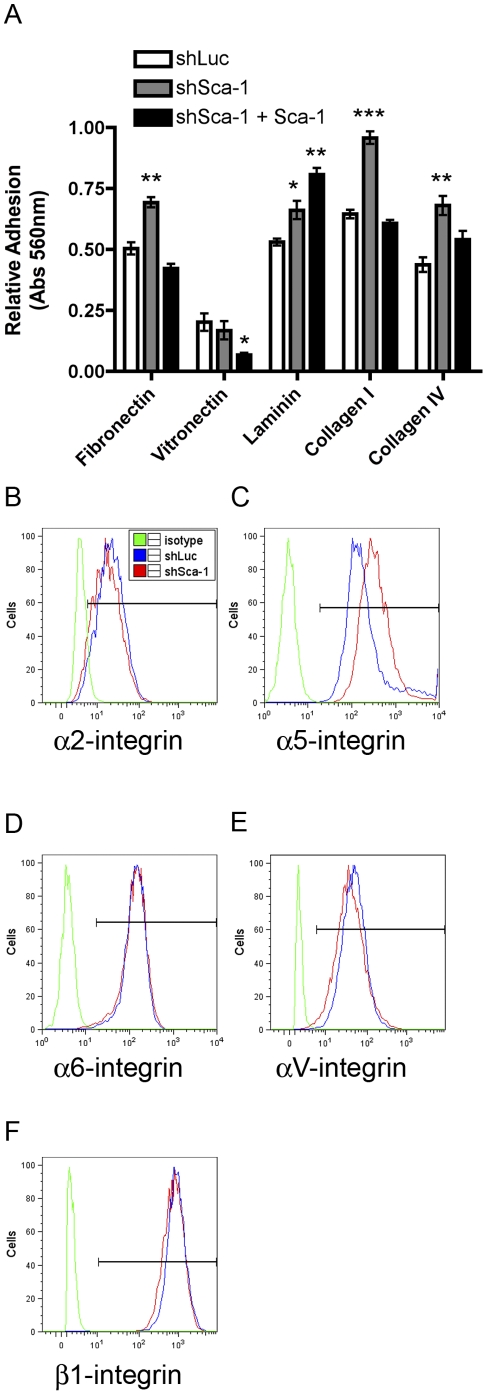
Sca-1 repression leads to increased adhesion to collagen I, collagen IV and fibronectin. Cell adhesion of 100,000 cells/well coated with fibronectin, vitronectin, laminin, collagen I or collagen IV comparing shLuc (open bars), shSca-1 (grey bars) and shSca-1+Sca-1 (black bars). (A). Relative adhesion was normalized to a BSA control, (* p<.05, ** p<.01, *** p<.001), mean±SEM of three experiments performed in triplicate. Flow cytometry analysis of integrin expression α2, α5, α6, αV, β1 (B–F, respectively) representative histograms.

We hypothesized that the delay in migration in the shSca-1 cells was attributed to alterations in cell adhesion. In order to determine if Sca-1 regulates cell adhesion, we evaluated the adhesion of the Wnt1-YL cells to a panel of extracellular matrix (ECM) proteins (collagen I, collagen IV, fibronectin, laminin, and vitronectin). shSca-1 cells showed increased adhesion to fibronectin, collagen I, collagen IV, and laminin compared to control cells (Figure 2A). In rescue experiments, adhesion of shSca-1+Sca-1 cells to collagen I, collagen IV, and fibronectin returned to levels similar to the shLuc control cells (Figure 2A). The increase in adhesion to laminin was enhanced in shSca-1+Sca-1 cells (Figure 2A). Additionally, each group exhibited relatively weak adhesion to vitronectin, however, the shSca-1+Scal-1 cells showed reduced adhesion compared to control cells (Figure 2A). These results suggest that delayed migration exhibited by shSca-1 cells may be due to increased cell matrix interactions. To investigate the possible cause for these altered adhesive properties, we evaluated the surface expression of integrins (receptors for ECM proteins) by flow cytometry. We analyzed a panel of integrins (α2, α3, α5, α6, αV, β1, β3, and β4) expressed in normal mammary epithelial cells. α2, α5, α6, αV and β1 were all expressed (Figure 2B–F); however, only α5-integrin showed a difference in expression level between the shSca-1 and control cells. α5-integrin expression increased 1.7 fold in shSca-1 cells (Figure 2C). Notably, α6 and β1, which bind laminin as a heterodimer were expressed at high levels as compared to their respective isotype controls (Figure 2D, F). Interestingly, these receptors have also been used to isolate cancer stem cells in p53^−/−^ mammary adenocarcinomas [11]. These data do not rule out the possibility that integrin activity rather than expression may be altered by Sca-1. Alternatively, integrins that were not evaluated may be aberrantly expressed accounting for the differences in cell adhesion.

### Repression of Sca-1 increases tumor propagation ability and accelerates tumor growth

We next determined if the alterations in migration and adhesion in Sca-1-deficient cells would influence tumor propagation and growth *in vivo*. To determine if the repression of Sca-1 effects tumor outgrowth, shSca-1 or shLuc cells were transplanted into the cleared mammary fat pad of 3–4 week old syngenic recipient mice, at concentrations ranging from 500–10,000 cells. Limiting dilution transplantation revealed that shSca-1 cells display a 9-fold increase in tumor propagating potential (1/654) as compared to shLuc control cells (1/5963; p>.001) (Table 1, [12]). A significantly greater number of shSca-1 tumors were observed at lower cell concentrations 2000-500 cells (Table 1).

**Table 1:** Repression of Sca-1 increases tumor outgrowth potential and tumor propagating cell frequency.

	Tumor Take Rate		
No. Cells Injected	shLuc (%)	shSca-1 (%)	p-value
**10,000**	9/10 (90)	11/11 (100)	ns
**5,000**	5/12 (42)	9/9 (100)	p<.01
**2,000**	4/10 (40)	13/14 (93)	p<.01
**1,000**	1/10 (10)	6/7 (86)	p<.01
**500**	1/11 (9)	7/13 (54)	p<.01
**Tumor Propagating Cell Frequency**	1/5963 cells	1/654 cells	

**Figure 3:**
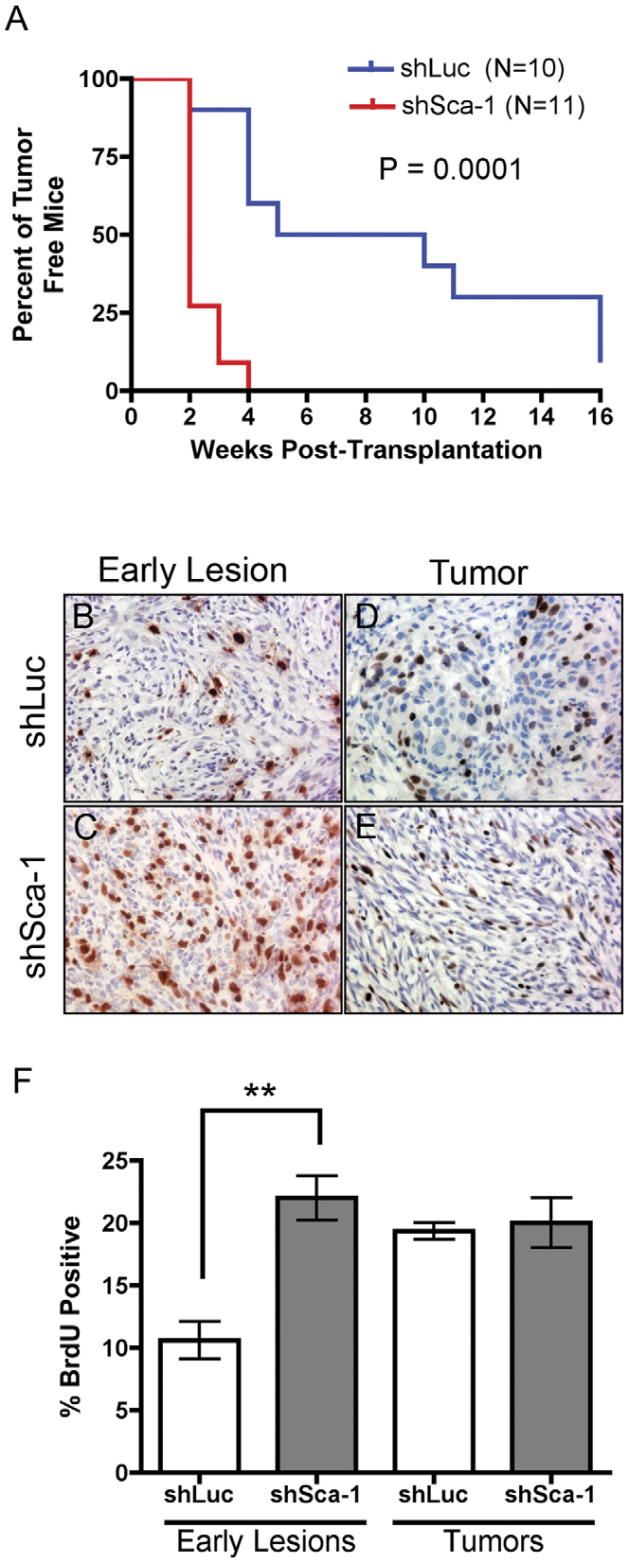
Repression of Sca-1 accelerates tumor growth by a transient increase in proliferation. Tumor latency plot comparing shLuc (blue line) and shSca-1 (red line) (A). BrdU staining of early lesions (B,C) and established tumors (D,E). Bar graph of BrdU staining of shLuc (open bars) and shSca-1 (grey bars) in early lesions and tumors (F) (** p<.01).

We subsequently studied tumor latency with injections of 10,000 cells, since both knockdown and control cell lines efficiently develop tumors at this concentration. shSca-1-derived tumors had a median latency of 2 weeks as compared to 5 weeks for shLuc-derived tumors (Figure 3A). To determine if the accelerated tumor development was due to increases in proliferation in the shSca-1 cells or increased cell death in the shLuc control cells, tumor sections were stained for BrdU and a TUNEL assay, respectively. This was investigated in both early histological lesions and in palpable tumors. When mammary fat pads were harvested 2 weeks following transplantation, and early lesions analyzed, 10% of the shLuc tumor cells were BrdU-positive in comparison to shSca-1 tumor cells in which 20% were BrdU-positive (Figure 3B–C, F). However, in established tumors this two-fold difference in proliferation was not observed, and approximately 20% of the cells were BrdU-positive cells in both groups (Figure 3D–F). Neither early lesions nor established tumors showed differences in cell death (Figure S1), suggesting that the difference in latency was attributed to a transient increase in proliferation in the early lesions. These data are consistent with the limiting dilution transplantation results and suggest that knockdown of Sca-1 influences tumor initiation in this model, but appeared to have little effect in established tumors.

**Figure 4:**
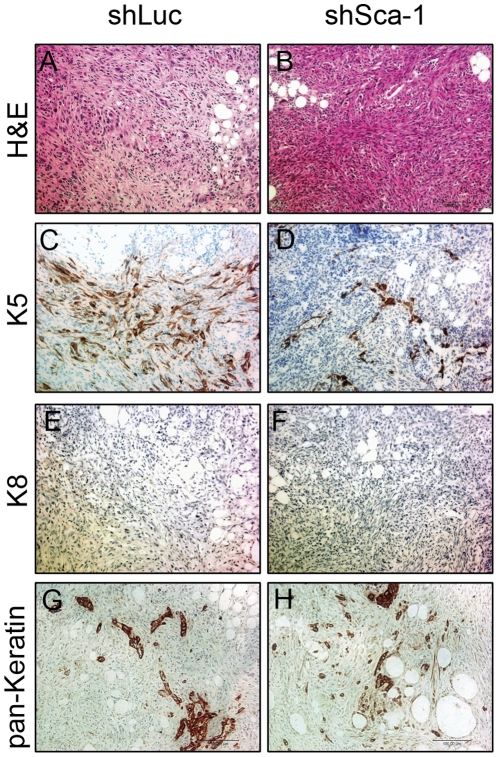
Wnt1-YL tumors express basal epithelial marker K5. Immunohistochemical staining of early tumor lesions of shLuc- and shSca-1-derived tumors. Hemotoxylin and eosin staining (A–B), Keratin 5 (C–D), Keratin 8 (E–F), pan-Keratin (G–H).

To determine the histological characteristics of these tumors, we performed immunostaining for mammary epithelial markers (K5, K8, pan-Keratin). Interestingly, hemotoxylin and eosin staining showed a mesenchymal (spindle-shaped) morphology distinctly different from the transgenic MMTV-Wnt1 tumors from which the cell line was derived. The tumors were positive for basal marker K5 (Figure 4), while only a small percentage of the cells were positive for luminal keratin marker K8, further suggesting a divergence from the parental MMTV-Wnt1 tumors, which expressed both basal and luminal keratins [8].

### Identification of differentially expressed genes

**Figure 5:**
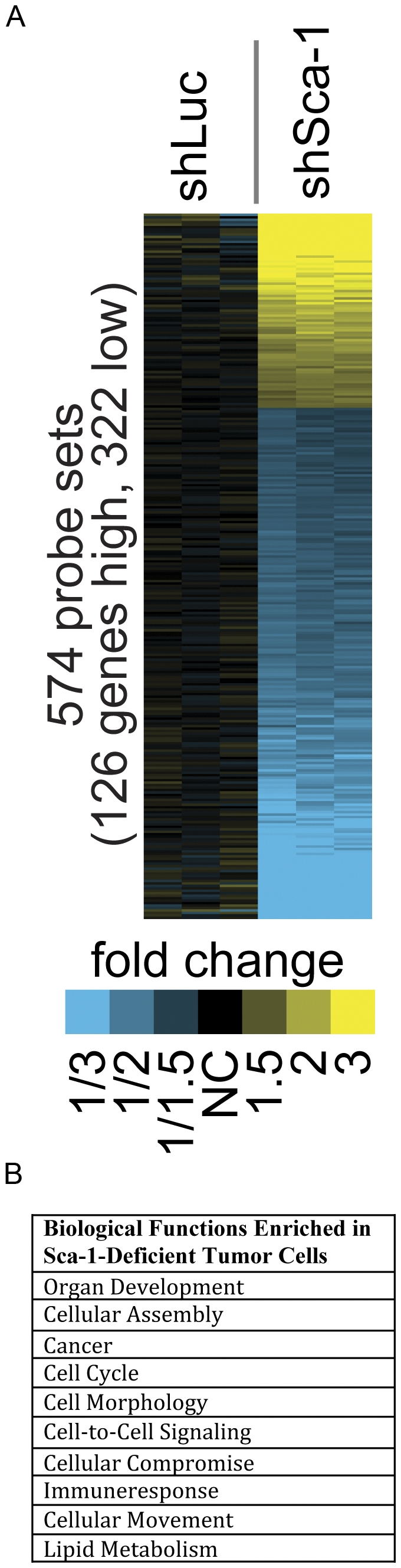
Gene expression analysis of shLuc and shSca-1 cells. Heat map of differentially expressed genes between shLuc and shSca-1 cells (A) up-regulated genes (yellow) and down-regulated (blue). Table of pathways enriched in Sca-1 deficient cells (B).

To determine the potential mechanisms by which Sca-1 regulates cell migration, adhesion, and tumor development, we performed an Affymetrix mouse genome 430A 2.0 array on cDNA comparing shLuc and shSca-1 from cells grown *in vitro*. The array identified 448 unique genes (574 Affymetrix probe sets) with p<0.01 and fold change >1.5 (Table S1). One hundred and twenty six genes were upregulated, and 322 genes were downregulated (Figure 5A). Importantly, Sca-1 was the only Ly6 family member on the chip that was significantly downregulated. Differences in gene expression of several genes were verified by qRT-PCR analysis (Figure S2). Repression of Sca-1 lead to the upregulation of several inflammatory chemokines: chemokine (c-c motif) ligand 2 (Ccl2), Ccl7, chemokine (c-c motif) ligand 5 (Cxcl5). Additionally, repression of Sca-1 altered the expression of genes involved in proliferation, cell movement, cell-cell signaling and cell organization (Figure 5B).

## Discussion

Sca-1 is widely accepted as a stem/progenitor cell marker in normal mouse tissues [3], [13]–[15]. However, Sca-1^eGFP/eGFP^ mice did not exhibit a reproducible phenotype on mammary gland development in our laboratory (unpublished data). Previous studies have shown that Sca-1 positive cells are expanded in Wnt/β-catenin induced mammary tumors [8], [9]. Additionally, a Ly6 family member, Ly-6D is upregulated in a variety of murine tumors and triple-negative breast cancers [16]. Despite these associations there is limited knowledge of functional role of Sca-1. Our findings indicate that Sca-1 plays an important role in mammary tumorigenesis as revealed using a novel cell line derived from MMTV-Wnt-1 mouse mammary tumors. First, Sca-1 promotes cell migration and affects cell adhesion to several ECM substrates in vitro. Second, Sca-1 regulates the frequency of tumor propagating cells and tumor cell proliferation in early lesions. These studies point to epithelial-ECM interactions as mediators of Sca-1 function; however, direct effects on downstream signaling and their relationship to tumor latency have not yet been determined.

There are several plausible explanations for our observations. First, Sca-1 may directly (or indirectly) interact with integrins modulating their ability to heterodimerize and bind ECM proteins, and/or modulating the strength of integrin-ECM interactions. The increased expression of α5-integrin in shSca-1 cells likely accounts for the increased adhesion to fibronectin via the α5β1 heterodimer. α5-integrin has been implicated as a suppressor of metastasis and α6-integrin promotes metastasis in breast cancer cell lines [17]. Since both of these integrins are expressed at similar levels in the Wnt1-YL cells, further investigation is required to fully define the relationship between Sca-1 and the role of integrins in this system. Next, Sca-1 may interact with non-integrin receptors such as growth factor receptors that cooperate with integrin signaling [18]–[20]. Alternatively, Sca-1 may alter interactions with cell surface receptors that act independently of integrin signaling. Additionally, Sca-1 may regulate the activation of MMPs leading to the release/activation of growth factors stimulating proliferation of tumor cells as observed in skeletal muscle cell regeneration [21].

The Wnt1-YL cells exhibited collective cell migration as a sheet in a scratch monolayer assay. In transwell migration assays, evaluating single cell migration across a porous membrane we did not show statistically significant differences in migration (data not shown). Furthermore, when the cells were seeded in matrigel (laminin-rich matrix) for a 3D morphogenesis/invasion assay the cells did not exhibit differences in terms of acini/colony formation frequency, size, morphology or invasive properties (data not shown). These observations suggest that Sca-1 is responsible for subtle changes in cell-cell and cell-ECM interactions in this cell line. Deciphering these subtleties in the context of cell migration and invasion may require further investigation of this cell line on matrices of single ECM substrates.

Interestingly, repression of Sca-1 alters chemokine expression, influencing the recruitment of inflammatory infiltrates. Since immune cells influence many processes including angiogenesis, cell invasion, matrix remodeling, interactions between tumor cells and the immune system have become of increasing interest in the past decade. Immune cells in both the innate and adaptive immune systems have proved to be important in tumor development and metastasis [22]–[25]. Chemokine secretion from shSca-1 cells may recruit immune cells with pro-tumor activities accounting for the accelerated tumor growth. Furthermore, Sca-1 not only regulates chemokine expression, but Wnt1-YL cells grown in culture show differential secretion of both cytokines and chemokines (data not shown). Also, insulin degrading enzyme (Ide-1), a protein that physically interacts with Sca-1 to regulate differentiation skeletal muscle cells [2], was down regulated in shSca-1 cells. Ide-1 catalyzes the degradation of mitogenic peptides attenuating proliferative signals. Reduction in this activity may also account for proliferative response seen the shSca-1 tumor development. Additionally, Fgf20, a Wnt/β-catenin target gene, was up regulated in response to Sca-1 repression. Cooperation between the Wnt/β-catenin and FGF signaling pathways has been reported in human cancers and our laboratory has previously shown a strong association leading to rapid proliferation upon simultaneous activation of these pathways [20]. Thus, Sca-1 potentially regulates multiple aspects of tumor development. The impact of these changes in mRNA expression needs to be determined with regard to protein expression and activity to better understand the role of Sca-1 in tumorigenesis.

Recently, Upadhyay and colleagues showed that Sca-1 inhibited TGF-β signaling by disrupting the heterodimerization of the TGF-β receptors and repressing expression of Gfd10, a TGF-β ligand, in a mammary adenocarcinoma cell line induced by medroxyprogestrone (MP) and 7,12-dimethylbenz(a)anthracene (DMBA) [26]. Their tumor outgrowth data indicate that repression of Sca-1 reduces tumorigenicity or outgrowth potential as observed in normal mammary epithelial cells. Similarly, another report shows delayed tumor development in MP/DMBA induced tumors in Sca-1 knock-out mice [27]. In this case, the delay in tumor development was attributed to the upregulation and activation of PPARγ. In contrast, our data indicate that Sca-1 may restrict cell growth. There are several explanations for these discrepancies. First, the tumors were developed under different conditions likely driven by different signaling pathways, which have been shown to yield very different tumor histopathologies [8]. Second, the relative level of Sca-1 on the cell surface is likely to govern how Sca-1 regulates signaling activities [1]. This may also account for the lack of overlapped genes in the microarrays when comparing the data of Upadhyay et. al. and our data set. Yuan et. al. only shared 15 genes in common with our data set with a p<0.01 and a fold change >1.5 (Table S2). These genes were all upregulated, but did not reveal an enrichment of a common functional pathway. Since the tumor cell models employed in the two studies were developed using different methods, it is likely that they express Sca-1 at different levels. Furthermore, it is unlikely that the efficiency of Sca-1 repression is the same as different shRNA constructs were used. Cell context no doubt plays an important role in influencing the effects of Sca-1 in tumors that may have been derived from very different cell lineages. For instance, CD24^high^/Sca1^−^ luminal mammary epithelial cells (MECs) do not express ER and PR and have increased *in vitro* progenitor activity in contrast to CD24^high^/Sca-1^+^ luminal MECs that are ER and PR positive with reduced in vitro progenitor activity [6]. Nevertheless, these studies highlight that Sca-1 likely regulates multiple cellular processes.

In conclusion, we provide evidence that Sca-1 regulates multiple cellular functions in mammary tumor cells. Our data highlight the importance of studying Sca-1 in the context of tumor development. To definitively differentiate the roles that Sca-1 plays in tumor initiation and tumor progression it will be necessary to use a conditional system in which Sca-1 can be knocked at various stages of tumor development. Additionally, it may be necessary to evaluate the role of Sca-1 in tumor subpopulations in models in which tumor-initiating cells are present. Further investigations along these lines will lead to a better understand of GPI anchored protein functions in tumors.

## Materials and Methods

### Ethics Statement

Mice were maintained in accordance with the National Institutes of Health Guide for the Care and Use of Experimental Animals with approval from the Baylor College of Medicine Institutional Animal Care and Use Committee (Animal Protocol: AN-504).

### Cell Line

The Wnt1-YL cell line was derived from an invasive carcinoma (ZD2508) of an MMTV-Wnt1 FVB/n mouse. The cells were grown in DMEM/F12 (Invitrogen) at ph 7.6, with 2% adult bovine serum (Gemini Bio-Products), antibiotic/antimycotic (Invitrogen), 5 µg/ml gentimycin (Sigma), 10 µg/ml insulin (invitrogen) and 5 ng/mL EGF (Invitrogen).

### Lentiviral Vectors

pLKO lentiviral vectors (Open Biosystems) with shRNA targeting Sca-1 were used to repress expression of Sca-1. The LeGO-Sca-1-iG2 was constructed by PCR amplifying Sca-1 cDNA from the pSport6-Sca-1 plasmid (Open Biosystems) using the follow primers: 5′-GCCGGGATCCCTGAGAGGAAGTTTTATCTGT-3′ and 5′-GCCGGAATTCTCAGAGCAAGGTCTGCAG-3′. The PCR product was digested BamHI and EcoRI cloned into the LeGO-iG2 expression vector.

### Lentiviral Transductions

293T-packaging cells were transiently transfected with pLKO-shRNA vectors (Open Biosystems), Gag-Pol and VSV-G plasmids using FuGENE 6 (Roche) according to the manufacturer's guidelines. Forty-eight hrs after transfection, virus-containing medium was collected from transfected 293T cells, filtered through a 0.45-mm syringe filter, and applied to Wnt1-2508 cells. The cells were spun at 300 *g* in a swinging platform rotor for 30 min. After 24 hrs, the lentiviral supernatant was removed from Wnt1-2508 cells and replaced with fresh medium. Forty-eight hrs later, cells were trypsinized and split at a low density with the addition of 4 mg/ml puromycin (Sigma) to select for transduced cells.

### Transplants

Clearance of the mammary fat pad and MEC transplantation procedures were performed as previously described [28]. Cells were trypsinized with 0.25% Trypsin-EDTA (Invitrogen) and counted using a Vi-CELL XR Cell Viability Analyzer (Beckman Coulter). The designated number of cells were washed and resuspended in Hank's balanced salt solution (Invitrogen). The cells were injected into the cleared inguinal fat pad of 3–4 week old FVB/n mice (Harlan). Tumors were allowed to develop for up to 16 weeks.

### Growth Curve

Cells were plated at 50,000 cells/well in 6-well plates and replenished with fresh medium every 48 hrs. Cells were trypsinized and counted every 24 hrs 4 days using a Vi-CELL XR Cell Viability Analyzer (Beckman Coulter).

### Adhesion Assay

10^5^ cells were seeded onto CytoMatrix™ Cell Adhesion Strips coated with BSA, Collagen Type I and IV, Fibronectin, Laminin, Vitronectin (Millipore) according to the manufacturer's guidelines. The cells were allowed to adhere for 1 hr at 37°C, non-adherent cells were washed off with PBS, and adherent cells were stained with 0.2% crystal violet. The relative attachment was determined by absorbance at 560 nm on a microplate reader and all samples were normalized to BSA coated wells. Statistical analysis was performed by one-way ANOVA followed by Tukey's multiple comparisons test.

### Migration Assay

Cells were grown to confluence in 6-well plates. A p1000 pipet tip was used to make a scratch down the center of each well. Pictures were taken on an inverted microscope (Zeiss) every 6 hrs for 24 hrs to evaluate migration across the scratch. Following the scratch, the cells were wash and refreshed with complete media. Axiovision software (Zeiss) was used to measure the distance across each scratch. For each experiment, 3 fields along the scratch of each well were analyzed in triplicate for each sample. A two-way ANOVA followed by Bonferroni tests was used to compare the mean at each time point.

### mRNA Real Time-PCR

cDNA templates were generated using a SuperScript II as previously described. Quantitative PCRs were run using SYBR Green reagent (Applied Biosystems) on a StepOnePlus thermocycler (Applied Biosystems), normalized to β-actin, and fold changes were calculated using the comparative CT (ΔΔCT) method using StepOne software v2.0.1 (Applied Biosystems). Primer sequences for Sca-1, Ccl2, Ccl7, Cxcl5, Mmp-9, and S100a8 were obtained from (Roche Applied Science).

### Flow Cytometry

Cells were trypsinized, trypsin was neutralized with culture medium containing ABS and centrifuged at 450× *g* for 5 min, and the cell pellet was resuspended in HBSS containing 2% ABS (HBSS+). Cells were counted and separated for labeling with antibodies. The cells were incubated primary antibodies for 20 minutes on ice, washed twice with HBSS+ and resuspended with HBSS+ containing Sytox Blue or Sytox Red (Invitrogen) to exclude dead cells. The cell suspensions were filtered using 40 µm filter (BD Falcon) and analyzed on a LSRII Fortessa (Becton Dickinson). The following primary antibodies were used: PE-conjugated (PE) rat anti-mouse Sca-1 (1∶100; BD Pharmingen), APC hamster anti-mouse/rat CD 29 (1∶100; BioLegend), FITC hamster anti-mouseCD49b (1∶100; eBioscience), PE mouse anti-human CD49c (1∶100; BD Pharmingen), PE rat anti-mouse CD49e (1∶100; BD Pharmingen), FITC rat ant-human CD49f (1∶100; BD Pharmingen), PE rat anti-mouse CD51 (1∶100; BD Pharmingen), Alexa Fluor 647 hamster anti-mouse/rat CD61 (1∶100; BioLegend), PE rat anti-human CD104 (1∶100; BD Pharmingen) and the corresponding isotype controls.

### Immunohistochemistry and Immunofluorescence

Mice were injected with 3 mg/mL BrdU (0.01 mL/g body weight) two hrs prior to sacrifice. Tumors were harvested and fixed in 4% paraformaldehyde for two hrs on ice. Tissues were embedded in paraffin blocks and 6–8 µm sections were cut for immunostaining. Sections were boiled in sodium citrate antigen retrieval buffer for twenty minutes. Sections were blocked with a 5% BSA, 0.05% Tween-20 in PBS for immunohistochemistry and in 10% goat serum in PBS for immunofluorescence. Primary antibodies were incubated at 4°C overnight. BrdU (1∶10; BD), K5 (1∶10,000; Covance), K8 (1∶5000; Univ. of Iowa), pan-Keratin (1∶5000; Sigma).

### Microarray

Total RNA was isolated from cells using TRIzol Reagent (Invitrogen) and then cDNA was made from total RNA with SuperScript II (Invitrogen) using random primers. cDNA was treated with RNase H (Invitrogen) to remove RNA. Microarray analysis was done with Affymetrix MG 430 2.0 chip. Statistical analysis was done with dChip software package (www.dChip.org), using PM-MM model and invariant set normalization. Differentially expressed genes were identified using two-sided t-test and fold change on log-transformed data. Java TreeView represented expression values as color maps [29]. Microarray data have been deposited into the Gene Expression Omnibus database (GSE30684) and followed MIAME requirements.

## Supporting Information

Figure S1 **Repression of Sca-1 did not alter cell death.** TUNEL staining of tumor sections (A–E). Positive control, DNaseI-treated shLuc tumor section (A). shLuc and shSca-1 early lesions (B, C) and tumors (D, E).
(TIF)

Figure S2 **qRT-PCR analysis of selected genes in shLuc and shSca-1 cells.** Relative mRNA expression of Sca-1, Mmp-9, S100a8, Cxcl5, Ccl2, and Ccl7 (A–F, respectively).
(TIF)

Table S1 **Differentially expressed genes in shSca-1 tumor cells.** Listed are statistically significant genes with p<0.01 and a fold change >1.5 in shSca-1 cells compared to shLuc control cells.
(XLS)

Table S2 **Upregulated genes associated with Sca-1 loss in tumors.** Listed are the statistically significant genes upregulated with p<0.01 and a fold change >1.5 in both shSca-1 cells and MP/DMBA tumors in Sca-1 knockout mice.
(XLS)
