# Supplementary figures and images for: Stem Cell Antigen-1 (Sca-1) Regulates Mammary Tumor Development and Cell Migration
Source: PLoS One. 2011 Nov 29;6(11):e27841. doi: 10.1371/journal.pone.0027841 (PMC3226565; doi:10.1371/journal.pone.0027841)

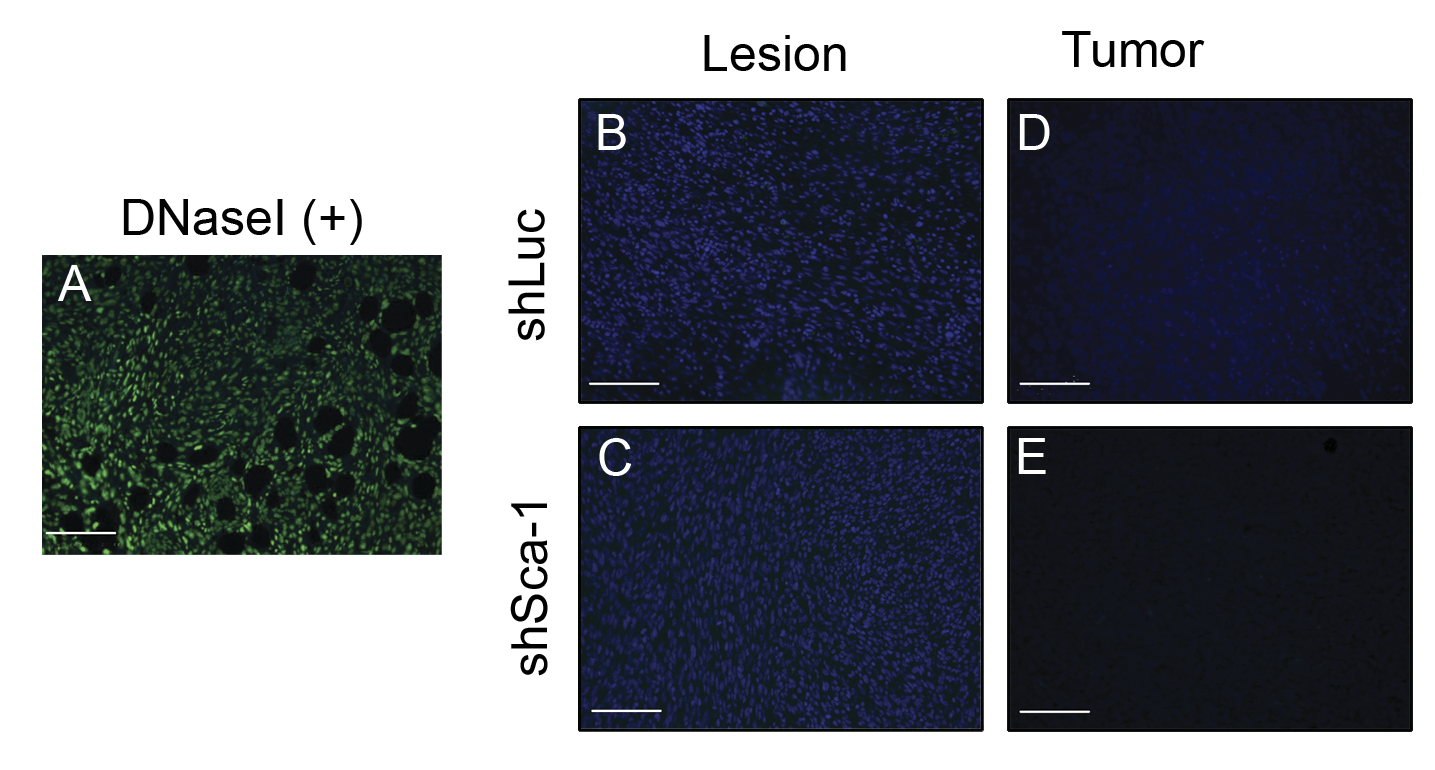

Supplement: Figure S1 — Repression of Sca-1 did not alter cell death. TUNEL staining of tumor sections (A–E). Positive control, DNaseI-treated shLuc tumor section (A). shLuc and shSca-1 early lesions (B, C) and tumors (D, E). (TIF) [file pone.0027841.s001.tif]

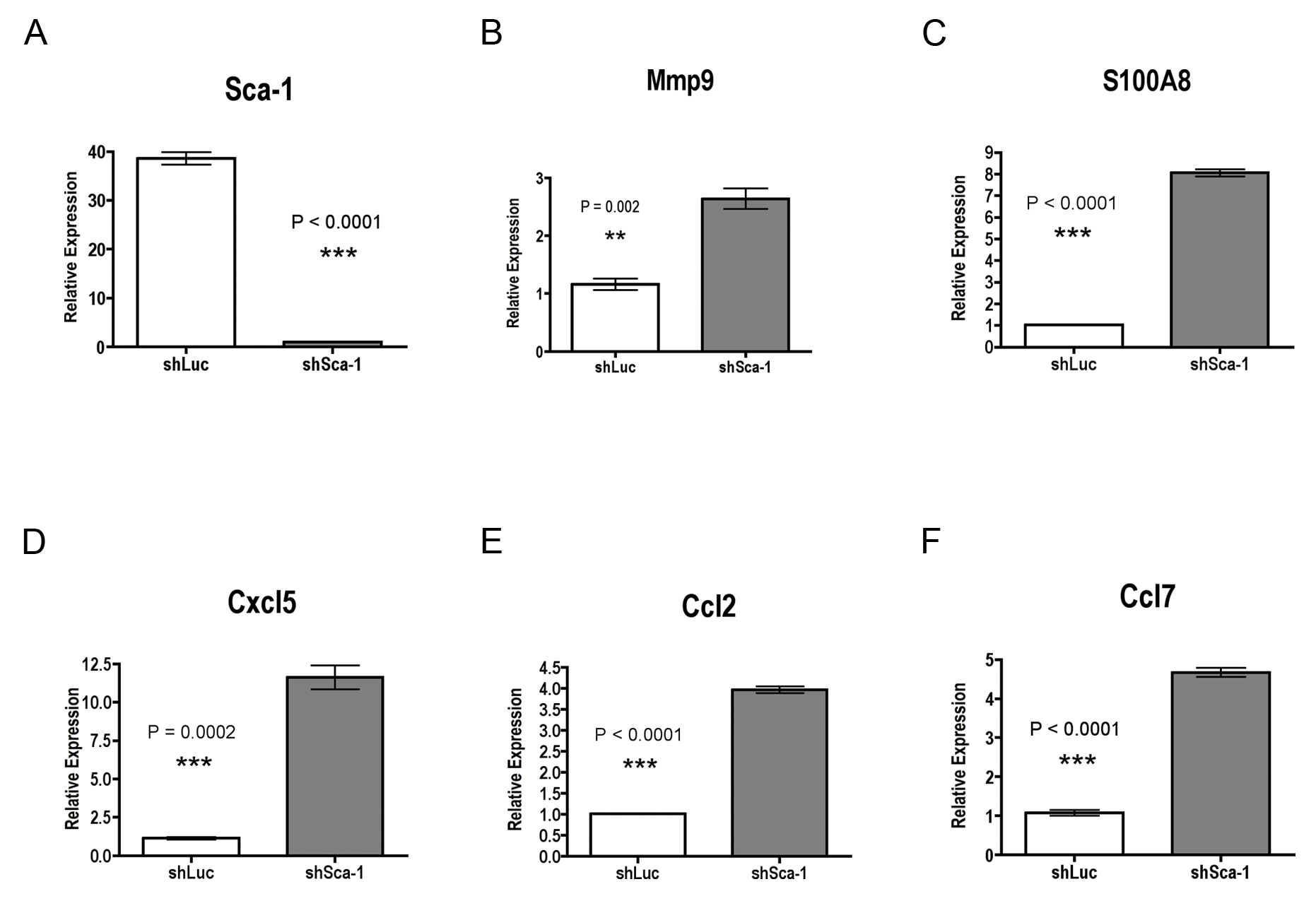

Supplement: Figure S2 — qRT-PCR analysis of selected genes in shLuc and shSca-1 cells. Relative mRNA expression of Sca-1, Mmp-9, S100a8, Cxcl5, Ccl2, and Ccl7 (A–F, respectively). (TIF) [file pone.0027841.s002.tif]
